# Hyperspectral Imaging Combined with Chemometrics Analysis for Monitoring the Textural Properties of Modified Casing Sausages with Differentiated Additions of Orange Extracts

**DOI:** 10.3390/foods12051069

**Published:** 2023-03-02

**Authors:** Chao-Hui Feng, Hirofumi Arai, Francisco J. Rodríguez-Pulido

**Affiliations:** 1 School of Regional Innovation and Social Design Engineering, Faculty of Engineering, Kitami Institute of Technology, 165 Koen-cho, Kitami 090-8507, Hokkaido, Japan; 2 RIKEN Centre for Advanced Photonics, RIKEN, 519-1399 Aramaki-Aoba, Aoba-ku 980-0845, Sendai, Japan; 3 Food Colour and Quality Laboratory, Facultad de Farmacia, Universidad de Sevilla, 41012 Sevilla, Spain

**Keywords:** hyperspectral imaging, texture, sausage core, orange extracts, modified natural casing

## Abstract

The textural properties (hardness, springiness, gumminess, and adhesion) of 16-day stored sausages with different additions of orange extracts to the modified casing solution were estimated by response surface methodology (RSM) and a hyperspectral imaging system in the spectral range of 390–1100 nm. To improve the model performance, normalization, 1st derivative, 2nd derivative, standard normal variate (SNV), and multiplicative scatter correction (MSC) were applied for spectral pre-treatments. The raw, pretreated spectral data and textural attributes were fit to the partial least squares regression model. The RSM results show that the highest R^2^ value achieved at adhesion (77.57%) derived from a second-order polynomial model, and the interactive effects of soy lecithin and orange extracts on adhesion were significant (*p* < 0.05). The adhesion of the PLSR model developed from reflectance after SNV pretreatment possessed a higher calibration coefficient of determination (0.8744) than raw data (0.8591). The selected ten important wavelengths for gumminess and adhesion can simplify the model and can be used for convenient industrial applications.

## 1. Introduction

Compared to conventional spectroscopic methods, hyperspectral imaging renders the measured reference parameters can be shown from spot to spot in samples [1,2,3,4,5]. Combined with the chemometrics analysis, it has been proved to be a powerful and emerging non-destructive technique that has been comprehensively applied to pork [6,7,8], lamb [9,10,11], beef [12,13,14,15], chicken [16,17,18], ham [19,20], and processed meat [3,5,21,22,23]. Wang and He (2019) non-invasively classified the Cantonese sausage degree using hyperspectral imaging, and the predictive accuracy can reach 100% [24]. The physicochemical and microbial attributes of bratwurst packaged in a pouch of 60 μm polyvinylidene chloride coated with polyamide (PVDC/PA) film after 20 days of storage (4 °C) were evaluated [25]. All of those illustrate that HSI is an emerging method to be widely applied to different types of meat products.

Sausage casing is the material used to encase sausage meat. It can be made from various materials, such as natural casings, synthetic casings, or collagen casings. Natural casings are typically made from the intestines of animals (e.g., sheep, pig (named as hog casing), or beef), while synthetic casings are made from materials such as cellulose, plastic, or fibrous materials. Collagen casings are made from collagen fibers, which are derived from the skin and connective tissue of animals. Sausage casings serve several purposes in the production of sausage. They provide a barrier between the sausage meat and the environment, helping to prevent spoilage and contamination. They also help to give the sausage its characteristic shape and size and can contribute to the texture and flavor of the final product. From the sausage quality point of view, the texture is an essential parameter to evaluate sausages, where the minced meat and fat were stuffed in the natural casings. Compared to artificial casings that are uniform, strong, flexible, and of hygienic quality, and natural casings are preferable to the sausage manufactories owing to their tenderness with a special bite [26]. Because the natural casings are susceptible to burst during production (such as handling, smoking, stuffing, cooking, and so on), there is a need to improve their properties to tolerate the high pressure generated during sausage production or post-cooking whilst maintaining the quality of the sausages. Feng et al. (2014) exploited the modified casings treated with a surfactant solution and slush salt with lactic acid and found less burst incidence occurred [27]. This is due to the porous structure, which releases pressure during stuffing or cooking. Nevertheless, this porous structure may render microorganisms easy to invade. There is a risk where lipid oxidation may occur. Therefore, it makes sense to add some natural additives to prolong the shelf life of this type of sausage.

It is well known that various bioactive compounds exist in citrus fruit peels and are useful and helpful for human beings [28]. Currently, waste orange peels (WOP) are not fully utilized and are either left in landfills as fertilizer or utilized as animal feedstuff [29]. Hesperidin, a common composition rich in citrus fruits, especially orange peels, showed its potential for therapeutics against COVID-19 in recent studies [30,31]. The effects of the addition of orange peel flour on the texture of sausage have been investigated [32]. The hardness of the sausages added with orange peel flour was significantly higher than the control group but showed better moisture and yield [32]. Díaz-Vela et al. (2015) studied the effects of the addition of cactus pear flour and pineapple peel flour to cooked sausages on the texture attributes during 20-day storage [33]. The softest samples were observed with the sausages containing cactus fruit peel flour, while the cohesiveness of samples with pineapple peel flour were different than those with cactus fruit peel, irrespective of the storage days [33]. In those studies, peels were used as the flour or fiber, and, up until now, few relevant studies has addressed evaluating the effects of orange peel extracts on the texture of sausages stuffed in modified hog casings. The objectives of the current study are to evaluate the effects of different combinations of surfactant solution and orange extracts addition on the textural properties of sausage cores by using hyperspectral imaging coupled with algorithms. Subsequently, important wavelengths will be selected for the sausage core texture with the different treated combinations.

## 2. Materials and Methods

### 2.1. Sample Preparation

Natural hog casing sections (30 cm), purchased from a local Japanese casing factory (Pakumogu.com, Niigata Prefecture, Japan), were desalted and modified in a solution composited of soy lecithin, soy oil, and orange extracts. A magnetic agitation was used to mix the solution and casing sections at 500 rpm and 25 °C. After the residence time, the casings (without rinsing) were put in salt and added with lactic acid for another residence time. Orange extracts were obtained from dried blade-milled Valencia sweet orange (*Citrus sinensis*) powder by Soxhlet extraction using 100% ethanol. The extracts from Soxhlet extraction were washed with a small amount of distilled water after leaving them in the fume hood overnight and transferring them to a filter paper. The crude orange extracts were obtained by drying naturally in a desiccator for three days. The detailed extraction procedures can be found in Feng et al. [29]. The effects of variables, soy lecithin (X_1_, SL), soy oil (X_2_, SO), residence time (X_3_, RT), the addition of orange extracts (X_4_, OE), and lactic acid (X_5_, LA), added in the slush salt, were studied by response surface methodology (RMS). The central composite design (CCD) was performed in Minitab 21.1 software (Kozo Keikaku Engineering Inc., Tokyo, Japan). The coded values and actual values of the independent variables were shown in Table 1. The CCD can provide the same quantity of information in all directions of the fitted surface by introducing star points. The star points were used to render the central composite design rotatable.

The center point was executed in sextuplicate to calculate the reproducibility of the method and a single run for each combination was conducted in Table 2 with a randomized order. In this way, the effect of unexplained variability in the observed responses owing to extraneous factors can be minimized. Hardness (*Y_h_*), springiness (*Y_s_*), gumminess (*Y_g_*), and adhesion (*Y_a_*) were the responses in this study. In order to develop the relationship between independent variables (*X_i_*; *i* = 1–5) and responses (*Y_n_*, n = h, s, g, a), a second-order polynomial equation was used to fit the experimental data:

(1) $$Y_{\left(n\right)}\;=\;\mu \;+\;\sum_{i=1}\mu_{i}X_{i\;}+\sum_{i=1}\mu_{ii}X_{i}^{2}+\sum_{i=1}\sum_{j=i+1}\mu_{ij}X_{i}X_{j}$$

where *μ*, *μ_i_*, *μ_ii,_* and *μ_ij_* are the constant, linear, quadratic, and interaction coefficients, respectively.

The accuracy of fitted models was estimated by determination coefficient (R^2^) and non-significant lack of fit.

Sausages were made using modified hog casings and natural hog casings. The composition of the sausage batter (total weight: 6966.80 g) was shoulder lean pork (57.70%), back fat (24.69%), salt (2.87%), sugar (1.72%), Chinese white wine (10.31%, ethanol content: 52% *v*/*v*), black pepper (1.06%), spicy pepper (0.61%), and seasonings (1.03%). A stuffing machine was employed to fill the well-mixed batter (curing 2 h at 4 °C) using modified casings and control natural casings. The sausages were twisted into sections and hung in the oven to dry for 24 h at 45 °C and aged at the temperature of 20 °C for an additional 48 h. Following this, the sausages were sectioned, sterilized, cut, and vacuum packaged. The packaged sausage sections were finally stored at 4 °C for sixteen days for HSI image capture and textural analysis.

### 2.2. Image Acquisition and Processing

The sausage cores were prepared with a diameter of 2.77 ± 0.16 cm and a height of 2.03 ± 0.16 cm. Images were captured by a laboratory visible 10-bit charged coupled device hyperspectral camera (NH-4-KIT, EBA Japan, Tokyo, Japan). The exposure time was 12.47 ms with push-broom line scanning. The flame rate was 100 fps and three halogen lamp lights were fixed beside the camera. A white sheet was employed to obtain an even light distribution and to avoid shadow. The camera spectral range was from 350–1100 nm divided into 151 bands. Samples were placed on a black sheet (to obtain a good contrast between the sample and background), perpendicular to the camera in a dark room with a room temperature controlled at 20 °C. A reflectance mode was used for imaging acquisition. Before measurement, a white reference with 100% reflectance was used to calibrate the HSI system. Dark reference was carried out by covering the camera lens with its opaque cap completely. The corrected images reference (R_correction_) was obtained via Equation (2)

(2) $$R_{\mathrm{correction}}=\frac{R_{1}-R_{2}}{R_{3}-R_{2}}$$

where *R*_1_, *R*_2_, and *R*_3_ are the raw, dark, and white reflectance images, respectively. A software named HSAnalyzer was employed to extract and analyze sample spectra. The version of this software is 1.2, produced by EBA Japan, Tokyo, Japan. The region of interest (ROI) of sausage core with different casing treatments was selected automatically: all pixels, which had reflectance higher than 0.06 at 735 nm, were considered for hardness, springiness, gumminess, and adhesive, respectively.

### 2.3. Textural Profile Analysis (TPA)

After HSI imaging acquisition, each meat core without casing was immediately put onto the center of a texture testing machine (EZ Test, Shimadzu Ltd., Kyoto, Japan) at room temperature of 20 °C. Two cycles of 50% compression with a 500 N aluminum cylindrical plunger (AL D36) load cell with a deformation rate of 1.0 mm/s were employed. The hardness, springiness, gumminess, and adhesion were recorded for the overall textural assessment. The parameter setting for conducting the textural profile analysis was based on Shin and Choi (2021) [34], Feng et al. (2014) [26], Herrero et al. (2008) [35], and the preliminary tests. Sausage textural analysis was conducted in triplicate.

### 2.4. Selection of Important Wavelengths

The weighted regression coefficients (BW) with large absolute values were selected as the important wavelengths. In this way, the model could be simplified and potentially improve its accuracy by eliminating the noise and redundancy information. A new simplified model was developed according to those selected wavelengths.

### 2.5. Model Development and Evaluation

The partial least square regression models with full and important wavelengths were used to establish the relationship between textural parameters and the spectra of the sausage cores with different casing modifications. Several spectral data pre-treatments, such as normalization, the 1st and 2nd derivatives, standard normal variate (SNV), and multiplicative scatter correction (MSC), were applied to improve the model’s performance. One-third of the samples (n = 11) were randomly chosen and used for the validation group, while the left two-thirds (n = 22) were used for the calibration group. The predictive ability was evaluated by the mean square error of calibration (RMSEC), validation (RMSEV), the determination coefficients of calibration (R_c_^2^), and validation (R_V_^2^).

### 2.6. Statistical Analysis

The simultaneous effects of SL, SO, RT, OE, and LA added in the slush salt were analyzed by Minitab 21.1 (shown in Table 3). The effects of different casing treatments on TPA were analyzed by Tukey’s HSD (honestly significant difference) ANOVA, as shown in Table 4 (one-way, IBM SPSS Statistics 28, Armonk, NY, USA).

## 3. Results and Discussion

### 3.1. Effects of SL, SO, RT, OE Addition, and Salt with LA on Textural Properties of Sausage Core

The simultaneous five effects on the textural properties of sausage cores were analyzed by response surface methodology. The highest R^2^ value of the regression model is the one developed for adhesion (77.57%), while the lowest value can only be 44.32%, developed by springiness. The lack of fits for models developed for all the textural parameters were insignificant (*p* > 0.05), indicating that those models were highly adequate. The predicted polynomial equations for the textural attributes, as in the uncoded units, are as follows:

(3) $$\begin{array}{c} Y_{h}\;=\;307.00\;-\;32.70\;X{}_{1}\;-\;17.60\;X{}_{2}\;-\;2.30\;X{}_{3}\;-\;290.00\;X{}_{4}\;-\;7.10\;X{}_{5}\;+\;1.44{\;X}_{1}^{2}\;+\;10.81{\;X}_{2}^{2}\;+\;0.01{\;X}_{3}^{2}+\;104.00{\;X}_{4}^{2}\;+\;\\ 2.56{\;X}_{5}^{2}\;+\;2.56\;X{}_{1}\;\times \;X{}_{2}\;+\;0.14\;X{}_{1}\;\times \;X{}_{3}\;-\;38.4\;X{}_{1}\;\times \;X{}_{4}\;+\;0.90\;X{}_{1}\;\times \;X{}_{5}\;+\;0.20\;X{}_{2}\;\times \;X{}_{3}\;+\;27.40\;X{}_{2}\;\times \;X{}_{4}\;-\;2.88\;X{}_{2}\;\times \;X{}_{5}\;-\;\\ 0.08\;X{}_{3}\;\times \;X{}_{4}\;-\;0.00\;X{}_{3}\;\times \;X{}_{5}\;+\;16.90\;X{}_{4}\;\times \;X{}_{5} \end{array}$$

(4) $$\begin{array}{c} Y_{s}\;=-12.08+0.29\;X{}_{1}+1.07\;X{}_{2}+0.00\;X{}_{3}+4.04\;X{}_{4}+1.08\;X{}_{5}+0.03\;X_{1}^{2}-0.13{\;X}_{2}^{2}+0.00{\;X}_{3}^{2}-3.74{\;X}_{4}^{2}-0.02{\;X}_{5}^{2}-\\ 0.03\;X{}_{1}\times \;X{}_{2}+0.00\;X{}_{1}\times \;X{}_{3}-0.04\;X{}_{1}\times \;X{}_{4}-0.03\;X{}_{1}\times \;X{}_{5}-0.01\;X{}_{2}\times \;X{}_{3}-0.37\;X{}_{2}\times \;X{}_{4}-0.00\;X{}_{4}\times \;X{}_{5}+0.01\;X{}_{3}\times \;X{}_{4}\\ -0.00\;X{}_{3}\times \;X{}_{5}-0.08\;X{}_{4}\times \;X{}_{5} \end{array}$$

(5) $$\begin{array}{c} Y_{g}\;=-9.66-0.51\;X{}_{1}-1.32\;X{}_{2}-0.02\;X{}_{3}+4.67\;X{}_{4}+1.13\;X{}_{5}+0.06{\;X}_{1}^{2}+0.19{\;X}_{2}^{2}-0.00{\;X}_{3}^{2}+3.49{\;X}_{4}^{2}-0.02{\;X}_{5}^{2}+\\ 0.15\;X{}_{1}\times \;X{}_{2}+0.00\;X{}_{1}\times \;X{}_{3}-0.57\;X{}_{1}\times \;X{}_{4}-0.00\;X{}_{1}\times \;X{}_{5}+0.01\;X{}_{2}\times \;X{}_{3}+0.11\;X{}_{2}\times \;X{}_{4}-0.03\;X{}_{2}\times \;X{}_{5}+0.04\;X{}_{3}\times \;X{}_{4}\\ +0.00\;X{}_{3}\times \;X{}_{5}-0.38\;X{}_{4}\times \;X{}_{5} \end{array}$$

(6) $$\begin{array}{c} Y_{a}\;=-15.31-0.95\;X{}_{1}+1.26\;X{}_{2}-0.01\;X{}_{3}-8.14\;X{}_{4}+1.67\;X{}_{5}+0.07{\;X}_{1}^{2}+0.14{\;X}_{2}^{2}-0.00{\;X}_{3}^{2}+4.68{\;X}_{4}^{2}-0.04{\;X}_{5}^{2}+\\ 0.02\;X{}_{1}\times \;X{}_{2}+0.01\;X{}_{1}\times \;X{}_{3}-0.96\;X{}_{1}\times \;X{}_{4}+0.00\;X{}_{1}\times \;X{}_{5}+0.01\;X{}_{2}\times \;X{}_{3}-0.05\;X{}_{2}\times \;X{}_{4}-0.13\;X{}_{2}\times \;X{}_{5}+0.04\;X{}_{3}\times \;X{}_{4}-\\ 0.00\;X{}_{3}\times \;X{}_{5}+0.33\;X{}_{4}\times \;X{}_{5} \end{array}$$

**Figure 1:**
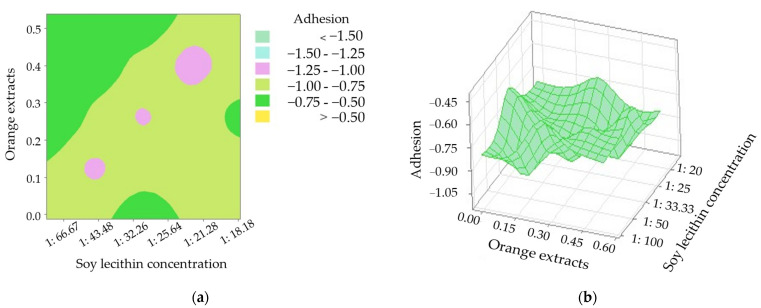
Adhesion of the sausage cores with modified casings as affected by orange extracts additions and concentration of soy lecithin. (**a**) two-dimensional contour plot; (**b**) three-dimensional surface plot.

For all the textural attributes of the sausage core, orange extracts (*X*_4_) showed the most important role according to the corresponding coefficient from Equation (3). Interactive effects of *X*_1_ × *X*_4_  on adhesion can be observed at a 5% significant level (Table 3). Based on the two-dimensional contour plot and the three-dimensional surface plot displayed in Figure 1, it can be discovered that adhesion decreased if a low SL concentration was associated with higher OEs. It is well known that hardness is defined as the peak force during the initial penetration cycle, while adhesion is defined as the negative area under the force-time curve following the first withdrawal [36]. It was reported that the increased adhesiveness may be due to the release of fat [36].

### 3.2. Spectra Overview

**Figure 2:**
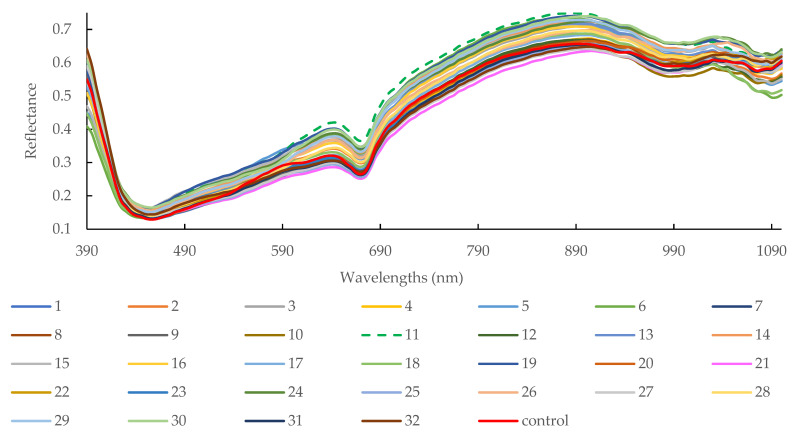
Mean reflectance spectral profiles of sausage cores with different casing treatments (i.e., different legend numbers) in the spectral range of 390–1100 nm.

Figure 2 illustrates the mean reflectance of sausages core with different modified casings. The reflectance of the sausage core with treatment 11 was higher (i.e., lower absorbance) than that of treatment 32. The average hardness of the sausage core with treatment, 11 (45.67 ± 5.69 N), was significantly lower than that with treatment, 32 (84.43 ± 1.01 N) (*p* < 0.05). The mean springiness of the treatment 11 (0.70 ± 0.38 mm) sample was higher than treatment (21 samples (0.48 ± 0.03 mm)) and control (0.46 ± 0.02 mm), although it was not significant at 5% level (Table 4, *p* > 0.05). Those indicate that sausage stuffed with the casing of treatment 11 possessed higher springiness with soft properties. Regarding adhesion, it represents the force required to overcome the stickiness or adhesion of a food product. The negative sign indicates that the force is acting in the opposite direction of the compression force used to deform the product. The higher the absolute value is, the stronger adhesion (i.e., sticky or slimy) will be. It can be found that control sample (−1.52 ± 0.38) and sample treated by treatment 5 (−1.51 ± 0.03) possessed significantly higher adhesion than that treated by treatment 12 (−0.44 ± 0.02, the lowest) (*p* < 0.05). There are several factors affect sausage’s adhesion, which includes meat composition, mixing grinding during sausage filling production, cooking temperature, casing types, additives, pH level, stuffing processing, and so on. In this study, the sausage filling was prepared in the same batch and stuffing and pre-cooking in the same behavior. The only difference may be due to the casing types and the pH change during the 16-d storage under different types of the modified casing. A pH level that is too high or too low can negatively affect the protein structure of the meat and finally lead to poor adhesion.

The absorbances are associated with the combinations of fundamental vibrations of C-H, N-H, O-H, and S-H functional groups [37]. For example, the slope shape between 600–700 nm is always related to oxymyoglobin formation. The third overtone of N-H stretching is related to a transmittance absorption band at 790 nm coupled with protein [38]. Subtle absorption at 780 nm and 980 nm may associate with the third and second overtones of O-H stretching, respectively, which may be relevant to water [39]. Absorption at 940 nm is related to C-H third overtone and relevant to fat [37].

### 3.3. Calibration Model Using Full Wavelengths

Table 5 displays the validation and calibration of textural attributes using full spectra. Only the adhesion of the PLSR model combined with 143 variables showed a comparably better-predicting capability than other textural properties. Compared with all the pre-treatments, R_c^2^_ (0.8744) and R_v^2^_ values (0.6837) after SNV treatment were higher than that of raw data (R_c^2^_ = 0.8591, R_v^2^_ = 0.5556), indicating the model improvement. The function of SNV is reported to remove variability in the reflectance spectra, resulting from light scattering [40]. Likewise, the function of MSC is to compensate for additive and multiplicative effects [41]. The individual discriminant factor analysis classified groups with HSI were assigned a quality deterioration index (QDI, composed of physicochemical, microbiological, and sensory analysis) to signify the quality of the packaged dry-cured sausages [2]. The R^2^ of calibration and validation produced by PLSR can achieve 0.99 and 0.96, respectively. The lower sample amounts may attribute to the low R^2^ values. It seems that only adhesion may comparably fit the PLSR model according to the R^2^ and RMSE values. As the hardness and springiness showed very low R_v^2^_ values and high RMSEV, only gumminess and adhesion were chosen for the following important wavelength selection.

### 3.4. Calibration Model Using Important Wavelengths

**Figure 3:**
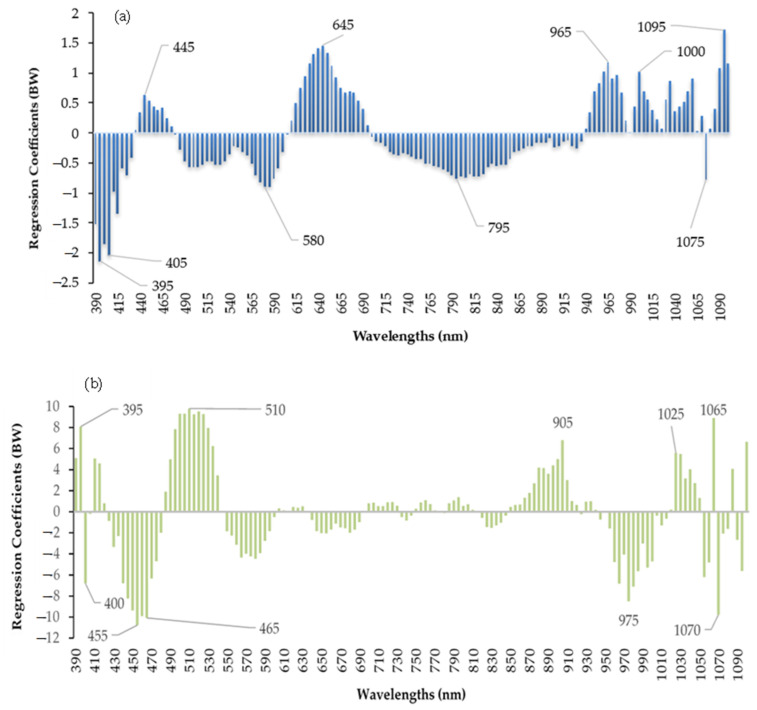
Selection of the important wavelengths for gumminess (**a**) and adhesion (**b**) from the PLSR model.

The gumminess of sausage refers to its texture, specifically the degree to which it is chewy or rubbery. A total of ten different important wavelengths were chosen for gumminess (395, 405, 445, 580, 645, 795, 965, 1000, 1075 and 1095 nm) and adhesion (395, 400, 455, 465, 510, 905, 975, 1025, 1065 and 1070 nm) using regression coefficients (Figure 3). The gumminess of sausage is defined as the energy required to chew semi-solid food until it can be swallowed, which is also influenced by a combination of factors, including the meat composition, casing, cooking temperature, mixing and grinding, additives, and pH level. Similar to adhesion, different casing treatments, and pH level evaluations may again attribute to the different gumminess values in the current study. Table 6 shows the predictive ability of the newly created models using the selected important wavelengths. If the accuracy of the new models at selected important wavelengths can be equivalent to that with full wavelengths, it provides useful information for the multispectral imaging system for real-time monitoring of the textural attributes of sausages core with different modified casings.

As depicted in Table 6, the R_c^2^_ value (0.5301) of gumminess improved slightly at the reduced model compared to the model using full wavelengths (0.4128). The R_v^2^_ value of gumminess cannot be performed with 2nd derivative treatment in the model with full wavelengths, while it can be performed using the reduced model. This may be due to the noise and pixel outliers’ removal. As for the adhesion, the prediction ability degraded using the selected wavelengths, but the models are simplified by lessening the full wavelengths.

## 4. Conclusions

In the current study, a hyperspectral imaging system was tested to investigate the probability of evaluating the textural properties of sausage core after 16 days of cold room storage. Sausage cores with treatment 11 modified casing were softer than that with treatment 32 modified casing sausage. Sausages with a treatment of five modified casings showed significantly higher adhesion than those treated by treatment of 12. Response surface methodology was capable to elucidate the relationship between different modification processing added with different concentrations of orange extracts and adhesion, with R^2^ of 77.57% and insignificant lack of fit. Ten important wavelengths were chosen for gumminess and adhesion, respectively, which provide useful information for a simple costless multispectral system development. This study shows how sausage textural properties respond to the different types of casing modification after adding the orange extracts, which could also be of practical use for future casing manufacturing.

## Figures and Tables

**Table 1:** Coded and uncoded physical values of operational parameters used in the CCD.

Point	Symbol	Coded (X_i_) Variable Level				
		Star	Low	Center	High	Star
		−2	−1	0	1	2
SL concentration ^1^	X_1_	1:90	1:45	1:30	1:22.5	1:18
SO concentration (%, *w*/*w*)	X_2_	0.625	1.25	1.875	2.5	3.125
RT (min)	X_3_	45	60	75	90	105
OE (%, *w*/*w*)	X_4_	0	0.12	0.26	0.4	0.54
LA (mL/kg NaCl)	X_5_	16.5	18	19.5	21	22.5

Note: SL: soy lecithin, SO: soy oil, RT: residence time, OE: orange extracts, LA: lactic acid. ^1^ SL: distilled water (*w*/*w*).

**Table 2:** Matrix of central composite design for each combination.

Samples	Surfactant Solution with OE				Salt with LA	
	SL Concentration (X_1_, *w*/*w*) ^b^	SO Concentration (X_2_, %, *w*/*w*)	RT (X_3_, min)	OE (X_4_, %, *w*/*w*)	LA (mL/kg NaCl, X_5_)	RT (X_3_, min)
1 ^a^	1:30	1.875	75	0.26	19.50	75
2	1:30	1.875	75	0.26	22.50	75
3	1:22.5	1.250	90	0.40	18.00	90
4	1:30	1.875	75	0.26	16.50	75
5	1:45	2.500	90	0.12	21.00	90
6 ^a^	1:30	1.875	75	0.26	19.50	75
7	1:45	2.500	60	0.40	21.00	60
8	1:90	1.875	75	0.26	19.50	75
9	1:30	1.875	105	0.26	19.50	105
10	1:30	1.875	45	0.26	19.50	45
11 ^a^	1:30	1.875	75	0.26	19.50	75
12	1:45	1.250	90	0.40	21.00	90
13	1:22.5	2.500	60	0.12	21.00	60
14	1:22.5	2.500	60	0.40	18.00	60
15	1:22.5	1.250	60	0.40	21.00	60
16	1:45	1.250	90	0.12	18.00	90
17	1:30	3.125	75	0.26	19.50	75
18	1:22.5	1.250	90	0.12	21.00	90
19	1:45	1.250	60	0.12	21.00	60
20	1:22.5	2.500	90	0.40	21.00	90
21	1:45	1.250	60	0.40	18.00	60
22	1:22.5	1.250	60	0.12	18.00	60
23	1:30	1.875	75	0.53	19.50	75
24	1:18	1.875	75	0.26	19.50	75
25	1:45	2.500	90	0.40	18.00	90
26 ^a^	1:30	1.875	75	0.26	19.50	75
27	1:22.5	2.500	90	0.12	18.00	90
28 ^a^	1:30	1.875	75	0.26	19.50	75
29 ^a^	1:30	1.875	75	0.26	19.50	75
30	1:45	2.500	60	0.12	18.00	60
31	1:30	1.875	75	0.00	19.50	75
32	1:30	0.625	75	0.26	19.50	75

Note: ^a^ Central points; ^b^ SL: distilled water (*w*/*w*), SL: soy lecithin, SO: soy oil, RT: residence time, OE: orange extracts, LA: lactic acid.

**Table 3:** Linear, quadratic, and interaction of the response variables of textural attributes.

Analysis of Variance	Df	Response Variables							
		Hardness (Y_h_)		Springiness (Y_s_)		Gumminess (Y_g_)		Adhesion (Y_a_)	
Source		Adj SS	F-Value	Adj SS	F-Value	Adj SS	F-Value	Adj SS	F-Value
Model	20	2195.12	0.87	0.52324	0.44	1.38276	1.29	2.19927	1.90
Linear	5	261.61	0.42	0.0711	0.24	0.17593	0.65	0.3688	1.28
Soy lecithin (X_1_)	1	10.34	0.08	0.00279	0.05	0.00831	0.15	0.10545	1.82
Soy oil (X_2_)	1	51.58	0.41	0.02982	0.50	0.00073	0.01	0.22761	3.94
Residence time (X_3_)	1	90.29	0.72	0.00156	0.03	0.01124	0.21	0.00472	0.08
Addition of orange extracts (X_4_)	1	54.83	0.44	0.03693	0.62	0.03286	0.61	0.0276	0.48
Lactic acid (X_5_)	1	54.57	0.43	0.00001	0.00	0.12279	2.28	0.00342	0.06
Square	5	759.30	1.21	0.33969	1.14	0.56663	2.11	0.74679	2.58
$X_{1}^{2}$	1	92.41	0.74	0.03333	0.56	0.16329	3.04	0.21651	3.74
$X_{2}^{2}$	1	523.37	4.17	0.07666	1.28	0.1684	3.13	0.08971	1.55
$X_{3}^{2}$	1	175.70	1.40	0.00137	0.02	0.01316	0.24	0.00012	0.00
$X_{4}^{2}$	1	113.35	0.90	0.14656	2.45	0.12764	2.37	0.22977	3.97
$X_{5}^{2}$	1	3.66	0.03	0.08727	1.46	0.08375	1.56	0.18835	3.26
2-Way Interaction	10	1174.21	0.94	0.11245	0.19	0.6402	1.19	1.08368	1.87
X_1_ × X_2_	1	50.52	0.40	0.00671	0.11	0.18471	3.44	0.00277	0.05
X_1_ × X_3_	1	81.41	0.65	0.00776	0.13	0.00134	0.02	0.24642	4.26
X_1_ × X_4_	1	550.21	4.39	0.00053	0.01	0.12008	2.23	0.34024	5.88 *
X_1_ × X_5_	1	36.25	0.29	0.03108	0.52	0.00096	0.02	0.00082	0.01
X_2_ × X_3_	1	56.47	0.45	0.04032	0.67	0.12035	2.24	0.06547	1.13
X_2_ × X_4_	1	89.04	0.71	0.01603	0.27	0.00143	0.03	0.00024	0.00
X_2_ × X_5_	1	116.42	0.93	0.00007	0.00	0.01488	0.28	0.23318	4.03
X_3_ × X_4_	1	0.41	0.00	0.00459	0.08	0.09796	1.82	0.088	1.52
X_3_ × X_5_	1	0.00	0.00	0.00083	0.01	0.00091	0.02	0.03097	0.54
X_4_ × X_5_	1	193.48	1.54	0.00453	0.08	0.09758	1.82	0.07558	1.31
Error	11	1380.02		0.65748		0.59121		0.63611	
Lack of Fit	6	1058.46	2.74	0.31435	0.76	0.22919	0.53	0.34281	0.97
Pure Error	5	321.56		0.34313		0.36202		0.2933	
R^2^ (%)		61.40		44.32		70.05		77.57	

Note: the value marked with * means significant at *p* < 0.05, and values with no mark mean not significant (*p* > 0.05). Df: degree of freedom. Adj SS: adjust sum of squares.

**Table 4:** Textural properties of sausage core with different modified casing treatments.

Sample	Hardness (N)	Springiness (mm)	Gumminess (N)	Adhesion (N s)
1	65.25 ± 18.75 ^ab^	0.51 ± 0.02 ^a^	0.97 ± 0.05 ^a^	−1.14 ± 0.01 ^abcdefg^
2	66.47 ± 7.42 ^ab^	0.53 ± 0.05 ^a^	0.54 ± 0.00 ^a^	−1.19 ± 0.17 ^abcdefg^
3	47.31 ± 10.05 ^ab^	0.96 ± 0.76 ^a^	0.59 ± 0.59 ^a^	−0.87 ± 0.32 ^abcdefg^
4	58.06 ± 1.67 ^ab^	0.48 ± 0.07 ^a^	0.98 ± 0.04 ^a^	−1.36 ± 0.06 ^bcdefg^
5	44.00 ± 12.58 ^b^	0.48 ± 0.03 ^a^	0.54 ± 0.05 ^a^	−1.51 ± 0.03 ^fg^
6	43.59 ± 11.75 ^b^	1.19 ± 0.44 ^a^	0.20 ± 0.04 ^a^	−0.79 ± 0.08 ^abcdefg^
7	66.84 ± 4.37 ^ab^	0.51 ± 0.07 ^a^	0.68 ± 0.37 ^a^	−0.93 ± 0.16 ^abcdefg^
8	79.66 ± 1.13 ^ab^	1.06 ± 0.62 ^a^	0.19 ± 0.04 ^a^	−0.51 ± 0.07 ^abcdefg^
9	78.96 ± 5.79 ^ab^	0.51 ± 0.06 ^a^	0.92 ± 0.06 ^a^	−1.18 ± 0.11 ^abcd^
10	62.32 ± 8.90 ^ab^	0.98 ± 0.20 ^a^	0.34 ± 0.17 ^a^	−0.75 ± 0.47 ^abcdefg^
11	45.67 ± 5.69 ^b^	0.70 ± 0.38 ^a^	0.57 ± 0.26 ^a^	−0.94 ± 0.04 ^abcdefg^
12	68.94 ± 14.13 ^ab^	0.76 ± 0.27 ^a^	0.17 ± 0.04 ^a^	−0.44 ± 0.02 ^a^
13	60.46 ± 4.33 ^ab^	0.44 ± 0.03 ^a^	0.31 ± 0.13 ^a^	−1.36 ± 0.57 ^defg^
14	54.05 ± 6.19 ^ab^	0.52 ± 0.02 ^a^	0.55 ± 0.04 ^a^	−1.50 ± 0.26 ^g^
15	62.99 ± 2.94 ^ab^	0.50 ± 0.02 ^a^	0.68 ± 0.37 ^a^	−0.97 ± 0.11 ^abcdefg^
16	57.13 ± 11.36 ^ab^	0.49 ± 0.06 ^a^	0.76 ± 0.03 ^a^	−1.04 ± 0.14 ^abcdefg^
17	71.07 ± 12.90 ^ab^	0.51 ± 0.22 ^a^	0.17 ± 0.04 ^a^	−0.80 ± 0.31 ^abcdefg^
18	74.55 ± 5.38 ^ab^	0.66 ± 0.25 ^a^	0.47 ± 0.37 ^a^	−0.74 ± 0.10 ^abcdefg^
19	61.27 ± 13.01 ^ab^	0.44 ± 0.02 ^a^	0.15 ± 0.08 ^a^	−0.65 ± 0.20 ^abcd^
20	70.65 ± 14.63 ^ab^	0.50 ± 0.00 ^a^	0.25 ± 0.02 ^a^	−0.99 ± 0.17 ^abcdefg^
21	64.11 ± 10.55 ^ab^	0.48 ± 0.03 ^a^	0.23 ± 0.02 ^a^	−0.65 ± 0.19 ^abcdefg^
22	67.95 ± 7.68 ^ab^	0.47 ± 0.02 ^a^	0.54 ± 0.33 ^a^	−0.90 ± 0.20 ^abcdefg^
23	77.12 ± 11.28 ^ab^	0.48 ± 0.02 ^a^	0.22 ± 0.04 ^a^	−0.58 ± 0.04 ^abcd^
24	56.24 ± 21.73 ^ab^	0.66 ± 0.07 ^a^	0.30 ± 0.08 ^a^	−0.71 ± 0.30 ^abcdefg^
25	66.46 ± 7.29 ^ab^	0.51 ± 0.02 ^a^	0.19 ± 0.02 ^a^	−0.56 ± 0.06 ^ab^
26	52.61 ± 4.10 ^ab^	0.49 ± 0.10 ^a^	0.40 ± 0.04 ^a^	−1.23 ± 0.15 ^abcdefg^
27	77.62 ± 1.73 ^ab^	0.57 ± 0.04 ^a^	0.38 ± 0.12 ^a^	−0.55 ± 0.03 ^abcdefg^
28	58.14 ± 10.65 ^ab^	0.54 ± 0.10 ^a^	0.75 ± 0.30 ^a^	−1.38 ± 0.01 ^abcd^
29	51.25 ± 1.80 ^ab^	0.67 ± 0.26 ^a^	0.67 ± 0.05 ^a^	−0.80 ± 0.30 ^cdefg^
30	55.71 ± 8.72 ^ab^	0.42 ± 0.01 ^a^	0.84 ± 0.11 ^a^	−0.92 ± 0.31 ^abcdefg^
31	60.31 ± 3.82 ^ab^	0.39 ± 0.03 ^a^	0.34 ± 0.39 ^a^	−0.61 ± 0.13 ^abcd^
32	84.43 ± 1.01 ^a^	0.52 ± 0.06 ^a^	0.31 ± 0.12 ^a^	−0.66 ± 0.25 ^abcdefg^
Control	64.47 ± 2.57 ^ab^	0.46 ± 0.02 ^a^	0.66 ± 0.60 ^a^	−1.52 ± 0.38 ^efg^

Note: values marked with different letters in a same column mean a significant difference (*p* < 0.05).

**Table 5:** Calibration and validation statistics for predicting textural attributes of sausage core using PLSR with full wavelengths range.

Parameters			Raw	Normalization	1st Derivative	2nd Derivative	SNV	MSC
Hardness (N)	Calibration group	R_c^2^_	0.6840	0.4760	0.4183	0.5772	0.4557	0.4552
		RMSEC (%)	7.0092	9.0264	9.5102	8.1079	9.2000	9.2039
	Validation group	R_v^2^_	Na	Na	Na	Na	Na	Na
		RMSEV (%)	9.5719	6.8142	5.6889	6.4465	6.1000	6.0803
Springiness	Calibration group	R_c^2^_	0.2083	0.9820	0.1485	0.2955	0.2653	0.2609
		RMSEC (%)	0.1782	0.2685	0.1848	0.1681	0.1716	0.1721
	Validation group	R_v^2^_	0.1785	0.2859	0.1267	Na	0.2014	0.1917
		RMSEV (%)	0.1615	0.1506	0.1666	0.2060	0.1593	0.1602
Gumminess (N)	Calibration group	R_c^2^_	0.4128	0.4005	0.9994	0.3245	0.6210	0.5919
		RMSEC (%)	0.1679	0.1697	0.0052	0.1801	0.1349	0.1400
	Validation group	R_v^2^_	0.3908	0.3462	0.1397	Na	0.5042	0.5091
		RMSEV (%)	0.2359	0.2444	0.2803	0.3360	0.2128	0.2117
Adhesion (N s)	Calibration group	R_v^2^_	0.8591	0.8494	0.9999	0.4825	0.8744	0.8451
		RMSEC (%)	0.1189	0.1229	0.0030	0.2279	0.1123	0.1247
	Validation group	R_v^2^_	0.5556	0.5742	0.3370	0.1706	0.6837	0.5949
		RMSEV (%)	0.2249	0.1964	0.2451	0.2742	0.1693	0.1916

Note: SNV: standard normal variate; MSC: multiplicative scatter correction; RMSEC: the root mean square error of calibration; RMSEV: the root mean square error of validation.

**Table 6:** Calibration and validation statistics for predicting textural attributes of sausage core using PLSR with selected important wavelengths range.

Parameters			Raw	Normalization	1st Derivative	2nd Derivative	SNV	MSC
Gumminess (N)	Calibration group	R_c^2^_	0.5301	0.4807	0.5063	0.5450	0.4984	0.4892
		RMSEC (%)	0.1502	0.1579	0.1540	0.1478	0.1552	0.1557
	Validation group	R_V^2^_	0.3620	0.4228	0.3862	0.3731	0.4354	0.4417
		RMSEV (%)	0.2414	0.2296	0.2368	0.2393	0.2271	0.2258
Adhesion (N s)	Calibration group	R_c^2^_	0.7746	0.7530	0.6877	0.7042	0.7311	0.6665
		RMSEC (%)	0.1504	0.1574	0.1770	0.1723	0.1643	0.1829
	Validation group	R_V^2^_	0.4427	0.4748	0.5440	0.3765	0.5248	0.4877
		RMSEV (%)	0.2247	0.2182	0.2033	0.2377	0.2075	0.2155

Note: SNV: standard normal variate; MSC: multiplicative scatter correction; RMSEC: the root mean square error of calibration; RMSEV: the root mean square error of validation.

## Data Availability

Data are contained within this article.
